# *Galleria mellonella*: A Novel Invertebrate Model to Distinguish Intestinal Symbionts From Pathobionts

**DOI:** 10.3389/fimmu.2018.02114

**Published:** 2018-09-19

**Authors:** Anna Lange, Andrea Schäfer, Annika Bender, Alexander Steimle, Sina Beier, Raphael Parusel, Julia-Stefanie Frick

**Affiliations:** ^1^Department for Medical Microbiology and Hygiene, Interfacultary Institute for Microbiology and Infection Medicine, University of Tübingen, Tübingen, Germany; ^2^Algorithms in Bioinformatics, ZBIT Center for Bioinformatics, University of Tübingen, Tübingen, Germany

**Keywords:** intestinal commensals, insect, innate immunity, symbiont, pathobiont, reactive oxygen species, antimicrobial peptides

## Abstract

Insects and mammals share evolutionary conserved innate immune responses to maintain intestinal homeostasis. We investigated whether the larvae of the greater wax moth *Galleria mellonella* may be used as an experimental organism to distinguish between symbiotic *Bacteroides vulgatus* and pathobiotic *Escherichia coli*, which are mammalian intestinal commensals. Oral application of the symbiont or pathobiont to *G. mellonella* resulted in clearly distinguishable innate immune responses that could be verified by analyzing similar innate immune components in mice *in vivo* and *in vitro*. The differential innate immune responses were initiated by the recognition of bacterial components *via* pattern recognition receptors. The pathobiont detection resulted in increased expression of reactive oxygen and nitrogen species related genes as well as antimicrobial peptide gene expression. In contrast, the treatment/application with symbiotic bacteria led to weakened immune responses in both mammalian and insect models. As symbionts and pathobionts play a crucial role in development of inflammatory bowel diseases, we hence suggest *G. mellonella* as a future replacement organism in inflammatory bowel disease research.

## Introduction

The intestinal microbiota plays a pivotal role in the development of intestinal inflammation and maintenance of homeostasis in humans and the respective mouse models. In this context bacteria of the intestinal microbiota beneficial for the host named symbionts, while commensals promoting intestinal inflammation in a genetically predisposed organism are named pathobionts (1). This is the case in inflammatory bowel disease (IBD), where certain commensals like *Escherichia coli* or *Klebsiella* genus, can accumulate and lead to disturbed immune responses (2, 3). In insects resident bacteria are also not always symbionts. It was demonstrated that *Gluconobacter morbifer* is pathobiotic in *Drosophila* and becomes “pathogenic” when the commensal microbiota composition is disturbed leading to deregulated intestinal immunity (4). Hence, insects might be suitable replacement organisms for discriminating intestinal mammalian symbionts from pathobionts.

Intestinal epithelial cells (IECs) mediate intimate contact between host and microbiota. IECs recognize bacterial components, so called microbe-associated molecular patterns (MAMPs), by pattern recognition receptors (PRRs) e.g., Toll-like receptors (TLRs) resulting in innate immune responses leading to the maintenance of homeostatic balance (5). Consequently, IECs secrete different molecules with antibacterial activity e.g., reactive oxygen species (ROS) (6), reactive nitrogen species (RNS) (7), antimicrobial peptides, (AMPs) or CCL20, an anti-inflammatory cytokine (5).

Recognition and effector components of the gut immune responses are evolutionary conserved (5).

The first line of intestinal innate immune defense is built up by the secretion of antimicrobial ROS and RNS, and is considered to be the most primordial innate immune response mechanism (8). Intestinal ROS can be produced primarily by NADPH oxidases (NOX), and under certain circumstances also by nitrate oxide synthase 2 (NOS2) (6); NOS2 mainly produces RNS (7). ROS and RNS are generated by phagocytes and respiratory and intestinal epithelial cells during infection to eliminate invading microbes (4, 7). However, an *in vivo* role of ROS relevant for host antimicrobial response in mammals needs to be elaborated, as most data involving ROS activity was obtained from *in vitro* experiments (4). ROS/RNS signaling requires detoxification, otherwise the substrates can be harmful for host cells and may lead to DNA, RNA or protein damage, or apoptosis (9). Antioxidant molecules like catalase, superoxide dismutase or glutathione S-transferase (GST) support detoxification (6). In *Drosophila* it has been shown that some bacteria can resist ROS and activate antimicrobial peptide production. Such AMP responses might complement ROS signaling under certain circumstances (4). Thus, gut innate immunity is further enhanced by AMPs, which have a critical role in reinforcing the intestinal barrier function and to prevent the uptake of bacteria (5). AMP expression is tightly regulated by the recognition of present microbes and AMPs are produced following PRR activation by IECs. However, the intestinal epithelium seems to mostly tolerate the intrinsic microbiota, and maintain a basal level of AMPs (5). In mammalian intestines AMPs are produced broadly in epithelial cells and in specialized cells in a constitutive or inducible manner. Among other AMP groups, defensins are central intestinal AMPs (10). Insects express AMPs as well and are often reported to be a very potent component of innate defense responses since insects lack adaptive immune responses (10, 11). Insects produce many AMPs simultaneously to potentiate their antimicrobial effect (11). The above mentioned strategies do not only contribute to fight invading pathogens but also help to balance the commensal microbiota composition and support maintenance of intestinal homeostasis (12).

Here we show that the larva of the greater wax moth *Galleria mellonella* is a suitable organism to distinguish between commensal symbiotic and pathobiotic intestinal microbiota members. In our study we chose an oral force-feeding model to mimic the natural route of colonizing a hosts' intestine with commensals. The *G. mellonella* larvae were administered with symbiotic *Bacteroides vulgatus* mpk, mediating an immune-balancing effect in mouse models of IBD, hence preventing from intestinal inflammation or the pathobiotic *Escherichia coli* mpk, which ignites inflammation (13, 14). We observed different hallmarks of *G. mellonella* innate immune responses like bacterial recognition, induction of ROS and AMP production. Furthermore, we analyzed similar components in mammalian innate immune responses *in vivo* and *in vitro* which are involved in responses toward pathogen infection or inflammatory responses. We demonstrated that *G. mellonella* can differentiate between symbiont and pathobiont homologous to mammalian systems based on the above mentioned innate responses (15).

## Materials and methods

### *Galleria mellonella* rearing

*Galleria mellonella* breeding was performed by transferring eggs laid by adult moths into wax moth substrate (22% corn grits, 22% wheatmeal, 17.5% beeswax, 11% skimmed milk powder, 11% honey, 11% glycerol, 5.5% dried yeast). Adult moths and larvae were kept in the dark at 30°C. Last instar larvae were used for experiments. Only pale and fast moving larvae were selected for experiments, to exclude interference with any background stress and immunity reactions during assay performance. Further, the larvae were weighed to ascertain than all larvae are in the similar last instar growth stage (180–200 mg).

### Bacterial strains and conditions

*Bacteroides vulgatus* mpk (13, 16) was grown at 37°C under anaerobic conditions. *B. vulgatus* was cultivated in liver broth for 2 days and subcultivated overnight in Brain Heart Infusion (BHI) medium. *Escherichia coli* mpk was grown in Luria-Bertani (LB) medium at 37°C under aerobic conditions. *E. coli* was grown overnight in LB broth and subcultivated for 2 h at 37°C on the next day prior to either force-feeding or stimulation of cell culture.

### Homology analysis

For homology assessment the BLASTX tool was used. *G. mellonella* sequences (Supplementary Table S1) were obtained from a published transcriptome dataset (17) and aligned to *Mus musculus* and *Homo sapiens* genomes.

As some of the relevant transcripts were only partial sequences, we did not align the fragments to distinct mouse or human protein sequences.

The decision whether proteins were homologous was based on commonly discussed criteria (18, 19). Proteins were considered homologous with 30% identity when completely covered and an *E*-value of 10^−6^. Further we considered proteins homologous if at least 70% of the sequence was covered with an *E*-value of above 10^−10^ and with at least 25% identity.

### Protein alignments

Protein sequences were aligned using MAFFT (20) and visualized using Unipro UGENE. Protein sequences were obtained from NCBI database: different *Lepidoptera* insect gallerimycin sequences [*Galleria mellonella* (AAM46728.1), *Spodoptera litura* (AEE37278.1), *Spodoptera exigua* (ADJ95798.1), *Spodoptera frugiperda* (AAP69838.1), *Spodoptera exigua* (AKJ54495.1), *Helicoverpa armigera* (ADR51151.1, partial), *Trichoplusia ni* (ABV68855.1), *Lonomia obliqua* (AAV91471.1), *Samia ricini* (BAG12297.1)], and mouse β-defensin 2 (BD-2) (P82020.1), human BD-2 (NP_004933.1), mouse (NP_058656.1), and human CCL20 (NP_004582.1) were used for the alignment. The consensus sequence was generated with the strict consensus type with a threshold of 90%. The agreements in hydrophobicity were highlighted.

The phylogenetic tree was created using Unipro UGENE and the PHYLIP Neighbor Joining tree building method with bootstrapping and majority rule (extended) consensus type.

### Cell culture

Transimmortalized mouse intestinal epithelial cells (IECs) (H2b)–mICcl2 cells–were originally derived from the small intestine of a transgenic mouse (21). Cells were grown in advanced Dulbecco's Modified Eagle Medium/Nutrient Mixture (DMEM)/F12 medium (Thermo Fisher) containing insulin-transferrin-selenium (ITS-G) (Thermo Fisher), 10% fetal calf serum (FCS) (Thermo Fisher), 5% Glutamine (Thermo Fisher), 10 nM epidermal growth factor (EGF) (Sigma), 1 nM triiodothyronine (Sigma), 50 nM dexamethasone (Sigma) at 37°C in 5% CO_2_/air atmosphere. The medium was used within 2 weeks of preparation to maintain the activity of growth factors.

Human SW480 cells were maintained in L-15 (Leibovitz) medium (Sigma) containing 2 mM Glutamine (Thermo Fisher) and 10% FCS (Thermo Fisher) at 37°C in normal air atmosphere.

Bone marrow-derived dendritic cells (BMDCs) were isolated from wild type C57BL/6J mice seeded and cultivated in petri dishes as described previously (22). Seven days after isolation cells were used for further *in vitro* stimulation experiments.

### Stimulation of miCcl2 cells, SW480 and BMDCs

mICcl2 cells and SW480 cells were seeded in Nunclon cell culture dishes for 24 h (Thermo Fisher). The next day mICcl2 and SW480 cells were stimulated with the bacteria MOI (multiplicity of infection) 10 for 0, 4, and 24 h whereas BMDCs were stimulated for 0.5, 2, and 4 h. PBS was used as mock stimulation control. Cells were harvested by scraping and centrifugation. Supernatants were used for ELISA and the remaining cell pellets were used for RNA extraction and analysis.

### Bacterial association of *Rag1^−/−^* mice

Six–Eight weeks old germ-free *Rag1*^−/−^ mice were orally administered with bacteria (*B. vulgatus* or *E. coli*) *via* drinking water. The bacteria were cultivated as described above and adjusted to 10^8^ bacteria per ml of sterile drinking water. The drinking water was replaced by sterile drinking water after 24 h. After 2 weeks of stable colonization, mice were transplanted intraperitoneally with 5 × 10^5^ naïve T cells isolated from wild type SPF C57BL/6J mice. After 4 weeks, mice were sacrificed for analysis. Cell scrapings were obtained from the distal ileum and the colon to extract RNA.

### Force-feeding of larvae with bacterial suspensions

The larvae were orally administered with 10^7^ bacteria. In order to force-feed the larvae the bacterial suspension was applied to their mouth using a syringe by inserting a blunt-ended needle between their mandibles. The syringe was placed into a microsyringe pump (World Precision Instruments) to ensure the accuracy of the suspension volume applied to each larva. The viable force-fed larvae were incubated in the dark at 37°C between 1 and 6 h. The larvae were snap-frozen after force-feeding. PBS-administered larvae were used as mock control to exclude the influence of stress responses potentially induced by the handling of the larvae.

### RNA extraction

*G. mellonella* larvae were incubated after force-feeding for 0–6 h. The individual larva was snap-frozen in liquid nitrogen and homogenized. Frozen homogenates were incubated in TRIzol (Sigma-Aldrich) for 1 h. After centrifugation the supernatant was mixed with 1-Bromo-3-Chloropropane (Sigma-Aldrich), incubated for 5 min at room temperature and incubated 10 min on ice. After centrifugation the upper layer was transferred into a new tube and RNA was precipitated with isopropanol. RNA pellets were washed with 70% ethanol. Dried RNA was resuspended with nuclease-free water containing RNasin Ribonuclease Inhibitor (Promega). The RNA contained the larval RNA as well as the bacterial RNA of the respective strain used for oral administration.

Mouse (mICcl2 cells, BMDCs, ileal, and colonic tissue) RNA was isolated from the cell pellets after centrifugation of the stimulated cell lines. For the isolation the RNeasy kit (QIAGEN) was used according to manufacturer's instructions.

### RT-PCR

5 μg of the isolated RNA was DNase-digested using DNA-free DNA Removal Kit (Thermo Fisher Scientific). Quantitative RT-PCR was performed using the QuantiFast SYBR Green RT-PCR Kit (Qiagen). Primers for quantitative RT-PCR were designed using NCBI Primer-BLAST design tool (Supplementary Table S2). Primers were checked for gene-specificity, i.e., fragment length, using PCR and agarose gel electrophoresis. In addition the primer efficiency was assessed; suitable primers had an efficiency of *E* = 2. In order to determine RNA gene expression, the ΔΔct method was used for calculations. Ubiquitin was used as housekeeping gene for *G. mellonella* RNA measurements, whereas actin was used as housekeeping gene in mouse RNA. Bacteria stimulated samples were normalized to mock controls.

### Quantification of bacterial load in force-fed *G. mellonella* larvae

Plasmid standards were generated by blunt-end cloning using pJET (Thermo Fisher Scientific) and the respective specific 16s PCR fragments of *E. coli* [Primer forward: GTTAATACCTTTGCTCATTGA, reverse: ACCAGGGTATCTAATCCTGTT (23)] or *B. vulgatus* (Primer forward: AACCTGCCGTCTACTCTT, reverse: CAACTGACTTAAACATCCAT (24)]. The concentration of the isolated plasmids was determined and the standard concentrations were prepared in 10-fold serial dilutions in a range of 100,000–10 copies. Groups of force-fed larvae were incubated for 1, 6, and 24 h. At the end of each timepoint the bacterial RNA was isolated as described above. cDNA was synthesized and concentration was measured using Qubit dsDNA High Sensitivity Assay (Thermo Fisher Scientific). For the qPCR measurement cDNA concentrations were adjusted to 5 ng per reaction and PCR was performed using QuantiFast SYBR Green PCR Kit (Qiagen). Bacterial copy numbers were determined by a standard curve for which log_10_ of standard copy numbers was plotted against ct-values.

### ELISA

The supernatants were collected by centrifugation after stimulation of the cultured cells. Human and mouse CCL20 (R&D Systems), mouse CD14 (R&D Systems), and human BD-2 (Arigo Biolaboratories) ELISAs were performed according to manufacturer's instructions. Absorbance was measured in absorbance reader (Tecan).

### Statistical analysis

Statistical analysis of the data was performed with GraphPad Prism 5 software. The distribution of the RNA and ELISA datasets was tested using the Kolmogorov-Smirnov method. RNA expression data was analyzed using the unpaired *t*-test, when the dataset was normally distributed. When datasets did not follow normal distribution, a nonparametric *t*-test was performed. ELISA datasets were analyzed using One-way ANOVA followed by Tukey's multiple comparison test. Differences were considered to be statistically significant if *P* < 0.05 (^*^P ≤ 0.05; ^**^P ≤ 0.01; ^***^P ≤ 0.001).

## Results

### Invertebrates and vertebrates share evolutionary conserved components of different innate immune responses

First we analyzed potential evolutionary relations between *Galleria mellonella*, mouse and human marker genes of oxidative stress responses (nitric oxide synthase (NOS), NADPH oxidase (NOX), and glutathione S-transferases (GST)), LPS recognition (apolipophorin, hemolin), and a defensin-like antimicrobial peptide (gallerimycin) on protein level. *G. mellonella* sequences were obtained from a published transcriptome dataset (17) and compared to *Mus musculus* and *Homo sapiens* genomes using BLASTX algorithm.

One transcript could be identifiie encoding for NOS in *G. mellonella*. Unfortunately, the full-length sequence of *G. mellonella* NOS protein was unavailable, but still the query coverage was approximatly 90% and identity greater than 40%. The transcript reaches *E*-values of 1 × 10^−6^ when aligned with BLASTX to the mouse genome, and *E*-values between 1 × 10^−7^ when aligned to the human genome (Table 1). We conclude that the sequences are homologous. Further we detected one transcript encoding for *G. mellonella* NOX. BLASTX alignment to the mouse and human genome revealed similarities and resulted in E-values below 10^−6^ (Table 1). However, homology cannot be confirmed resolutely as the query coverage was only 64% with 34% identity for mammalian NOX proteins and 62% with 25% identity for mouse and human DUOX proteins.

**Table 1:** BLASTX analysis of nitric oxide synthase (*Nos*) and NADPH oxidase (*Nox*) genes.

***G. mellonella***	***Mus musculus***				***Homo sapiens***			
**Transcript**	**BLAST Hit**	**Identity**	**Query coverage**	***E* value**	**BLAST Hit**	**Identity**	**Query coverage**	***E* value**
Nitric oxide synthase	Nitric oxide synthase	42%	94%	1 × 10^−06^	Nitric oxide synthase	40%	90%	1 × 10^−07^
NADPH oxidase	NADPH oxidase 4	34%	64%	4 × 10^−79^	NADPH oxidase 4	34%	64%	4 × 10^−79^
	Duox2 protein	25%	62%	3 × 10^−30^	Dual oxidase 1	24%	62%	7 × 10^−26^

The transcriptome analysis revealed 19 different available GST variants (17). We found matches with mammalian GST proteins with *E*-values below 10^−6^ (Supplementary Table S3) after BLASTX alignments, but query coverage sometimes even lower than 30%. Only five GST transcripts had query coverage of above 70%. Those proteins were found to have reasonable *E*-values above 10^−10^ and identity of more than 25%.

In the available transcriptome dataset we could further identify relevant LPS-binding molecules. Specifically, five different transcripts encoding for the LPS recognition molecule apolipophorin were detected as well as two transcripts of hemolin, another LPS-binding opsonin. However, none of those transcripts were shown to have similarity with relevant mouse or human proteins after BLASTX analysis.

**Figure 1:**
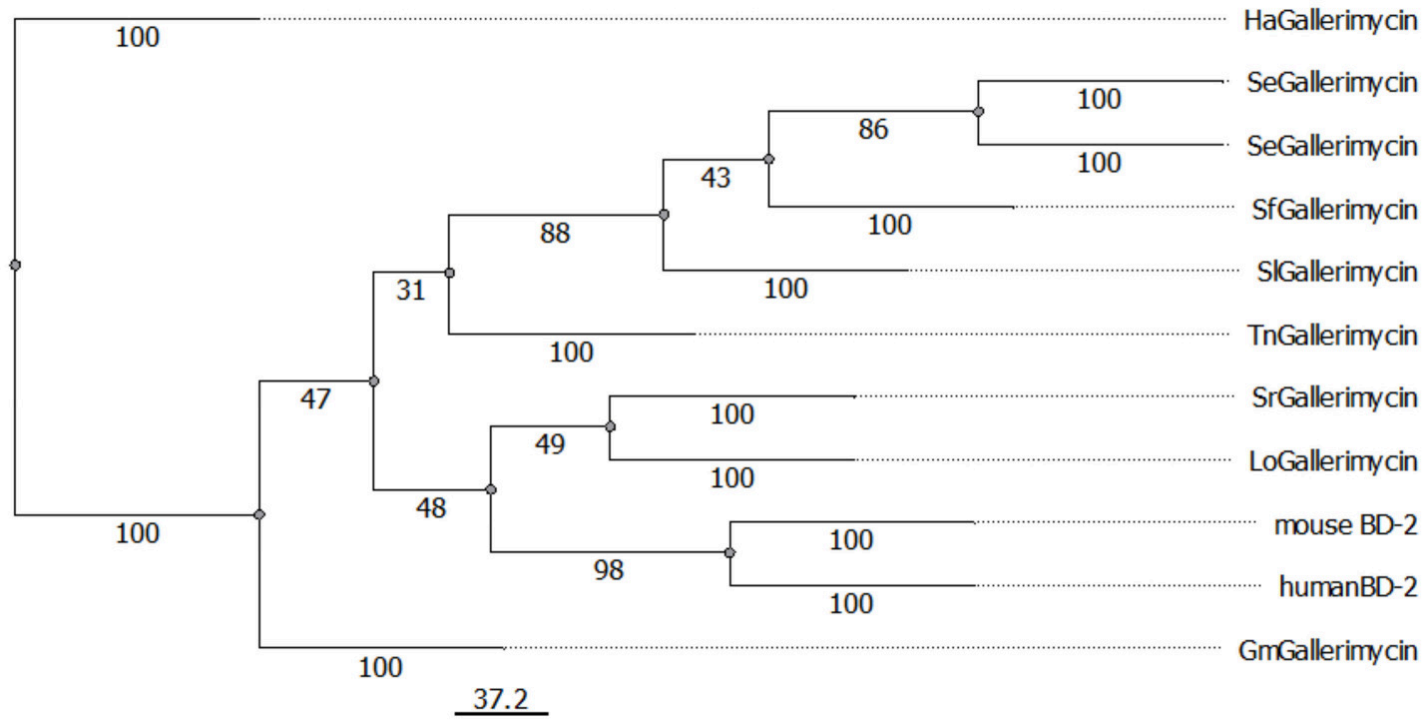
Display of evolutionary relation between lepidopteran gallerimycins and mouse and human β-defensin 2 proteins. Phylogenetic relations among gallerimycin sequences of various lepidopteran insects (Gm: *Galleria mellonella*, Ha: *Helicoverpa armigera*, Lo: *Lonomia obliqua*, Sr: *Samia ricini*, Se: *Spodoptera exigua*, Sf: *Spodoptera frugiperda*, Sl: *Spodoptera litura*, Tn: *Trichoplusia ni*) and the mouse and human BD-2 sequences were displayed using PHYLIP Neighbor Joining method. The scale bar for branch length is shown below the tree. NCBI reference IDs for each sequence is available in the material and methods section.

For *G. mellonella* defensin-like antimicrobial peptide (AMP) gallerimycin, a full-length protein sequence was available in the NCBI database and observed possible similarities with mouse and human β-defensin-2 (BD-2) in more detail. We also integrated other insect gallerimycins available from NCBI in our analysis. Different sequences were chosen from the other *Lepidoptera* order in which *G. mellonella* also belongs. A protein alignment revealed similar regions of hydrophobic (red) and hydrophilic (blue) areas in all sequences independent from the organism (Supplementary Figure S1). This suggests that the similar regions account for similar functions such as capabilities to bind other interacting molecules. Further we built a phylogenetic tree with the insect gallerimycin protein sequences and the mouse and human BD-2 sequences (Figure 1). The mouse and human BD-2 peptides showed the closest relation to each other. Interestingly, gallerimycin of the giant silkworm moth *Lonomia obliqua* and the ailanthus silkmoth *Samia ricini* were closer related to mBD-2 and hBD-2 compared to gallerimycin of the other *Lepidoptera* insects.

Additionally, we included the antimicrobial cytokine CCL20 to our analysis, because it shares structural homology with BD-2 (5). We aligned *G. mellonella* gallerimycin, mBD-2, hBD-2, mouse and human CCL20. Interestingly, as shown in the previous alignment, similar hydrophobic (red) and hydrophilic (blue) distribution of amino acids could be observed (Supplementary Figure S2).

### *G. mellonella* and mice show increased production of LPS-binding recognition molecules in response to pathobiont

In previous work we showed that the pathobiont *E. coli* mpk induces colitis in *Il2*^−/−^ mice, whereas the symbiont *B. vulgatus* mpk maintains homeostasis (13). Intestinal commensals can either be symbionts or pathobionts. This distinction becomes crucial in inflammatory bowel disease (IBD) research as IBD comes along with increased levels of pathobionts (2). *Bacteroides* species on the other hand are reported to exhibit different immunomodulatory effects (14, 25).

We monitored if the symbiont *B. vulgatus* mpk or the pathobiont *E. coli* mpk were differentially recognized. Both bacteria are Gram-negative and contain Lipopolysaccharides (LPS). Thus, regulation of the LPS-binding molecules apolipophorin (*ApoIII*) and hemolin in *G. mellonella* and an analogous component of mammalian cells, CD14, which is an important LPS-binding molecule involved in microbe recognition (26), was observed.

**Figure 2:**
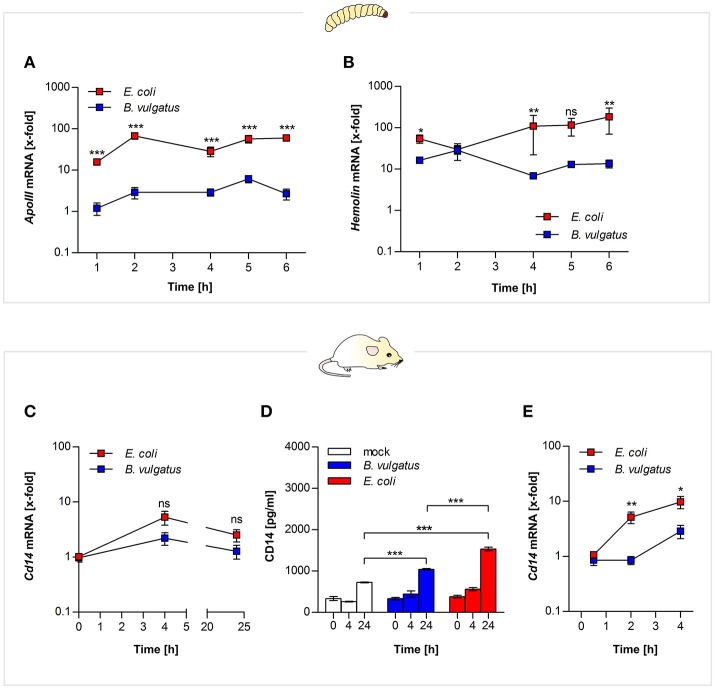
MAMP recognition by both vertebrate and invertebrate pattern recognition receptors. Larvae were force-fed with bacteria and immune responses were observed over time and RNA was isolated from individuals. Gene expression of LPS recognition molecule apolipophorin **(A)** and opsonin hemolin **(B)** was measured from *G. mellonella* RNA (*n* = 8). Mouse mICcl2 cells were stimulated with *B. vulgatus* or *E. coli* for 0, 4, and 24 h. Gene expression of *Cd14* an LPS receptor molecule was determined in the isolated RNA (*n* = 5) **(C)**. Secretion of CD14 in mICcl2 cell culture supernatants was analyzed by ELISA (*n* = 3) **(D)**. *Cd14* gene expression was further detected in supernatants of BMCDs of C57BL/6 mice stimulated with *B. vulgatus* or *E. coli* for 0.5, 2, and 4 h. (*n* = 5) **(E)**. Data represent the geometric means ± SEM.

*E. coli* administration of *G. mellonella* larvae lead to stronger gene expression of both *ApoIII* and hemolin after 2 h and remained elevated over time, compared to administeration with the symbiont *B. vulgatus* (Figures 2A,B).

Likewise small intestinal mouse mICcl2 cells stimulated with *E. coli* provided higher *Cd14* gene expression after 4 h (Figure 2C). The corresponding cell culture supernatants of the mICcl2 cells were assayed for secretion of CD14 protein by ELISA. CD14 levels were significantly enhanced in supernatants of *E. coli*-stimulated cells after 24 h compared to *B. vulgatus* stimulation (Figure 2D). In compliance with this, we reported about increased intestinal *Cd14* expression levels in *E. coli*-associated gnotobiotic *Il2*^−/−^ mice compared to *B. vulgatus*-associated gnotobiotic mice (13).

In addition, we stimulated BMDCs of C57BL/6 mice with either *E. coli* or *B. vulgatus* for 0.5, 2, and 4 h. Gene expression levels of *Cd14* were significantly higher in *E. coli*-stimulated cells compared to *B. vulgatus* stimulation similarly demonstrated in small intestinal cells (Figure 2E).

### Increased ROS-related gene expression by intestinal commensals in insect and mammalian hosts

Rapid environmental changes may force an organism to quickly adapt by triggering a cytoprotective survival program. This program can lead to increased production of reactive oxygen and nitrogen species (ROS/RNS) and can cause oxidative stress (27). In previous work, we already indicated a higher accumulation of ROS in *E. coli*-stimulated bone marrow-derived dendritic cells (BMDCs) compared to *B. vulgatus* stimulation (14).

**Figure 3:**
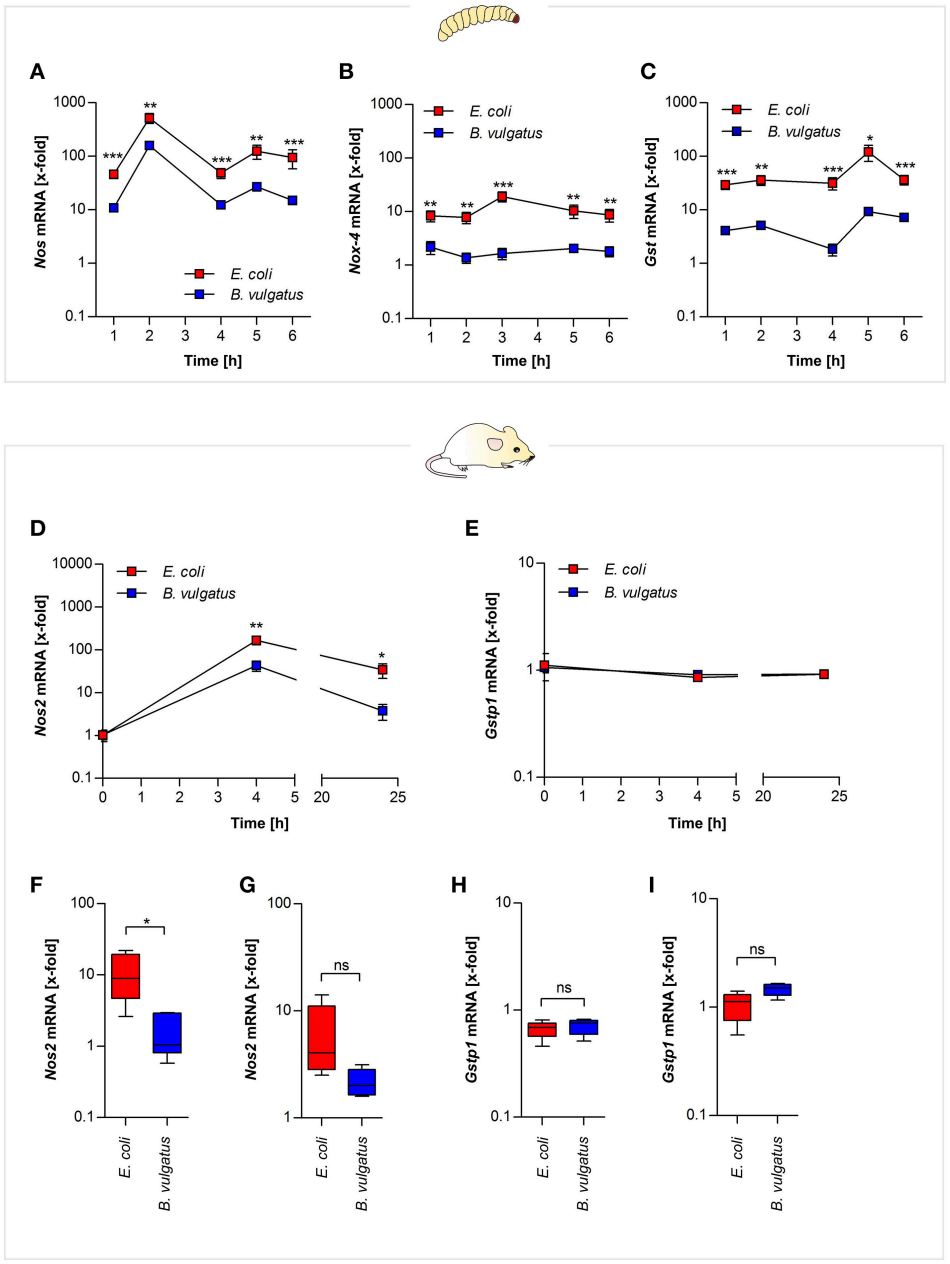
ROS- and RNS-related gene expression after symbiont or pathobiont challenge. *E. coli* and *B. vulgatus* orally administered larvae were monitored over time. Gene expression of ROS defense response markers was investigated. *Nos* **(A)**, *Nox-4* **(B)**, and *Gst* **(C)** gene expression was measured in RNA isolated from whole individuals (*n* = 8). RNA was isolated from mouse mICcl2 cells stimulated with *B. vulgatus* or *E. coli* for 0, 4, and 24 h. *Nos2* **(D)** and *Gstp1* **(E)** gene expression was determined (*n* = 5). Gnotobiotic *Rag1*^−/−^ mice were associated with *B. vulgatus* or *E. coli* for 4 weeks. *Nos2* gene expression was determined from RNA isolated from ileal **(F)** and colonic **(G)** sections. In addition, ileal **(H)** and colonic **(I)** *Gstp1* gene expression was measured (*n* = 5). Data represent the geometric means ± SEM.

In order to test whether different commensals cause different ROS-related gene expression in *G. mellonella* and gnotobiotic mice, *E. coli* or *B. vulgatus* were orally-administered to both organisms. Gene expression of *Nos* and *Nox* was determined from RNA isolated from either whole larval individuals or from ileal and colonic sections of bacteria-administered mono-associated *Rag1*^−/−^ mice. Oral administration of *E. coli* to *G. mellonella* induced significantly higher *Nos* gene expression compared to *B. vulgatus* administration after 2 h and *Nox-4* expression after 3 h (Figures 3A,B). After 5 h of bacterial administration, when initial *Nos* and *Nox-4* gene expression levels had decreased, the larvae induced *Gst* gene expression (Figure 3C).

To investigate further if mouse intestinal cells display similar expression of the homologous ROS-responsive *Nos2* gene, we used small intestinal mICcl2 cells. The cells were stimulated with *E. coli* and *B. vulgatus* for 0, 4, and 24 h and *Nos2* expression was determined by quantitative PCR. *Nos2* Expression of *E. coli*-stimulated cells was significantly increased after 4 and 24 h (Figure 3D). *Gstp1* gene expression on the other hand was not regulated at any time point (Figure 3E).

The assessment of *in vivo* relevance of oxidative stress in response to symbiont or pathobiont association was performed in Rag1^−/−^ gnotobiotic mice. After 4 weeks of mono-association with either *E. coli* or *B. vulgatus Nos2* gene expression was determined in ileal and colonic sections. *Nos2* gene expression in response to *E. coli*-association was more pronounced compared to *B. vulgatus* association (Figures 3F,G), whereas *Gstp1* expression was not induced (Figures 3H,I).

### Pathobionts activate stronger antimicrobial peptide responses

Recognition of microbes by sensing their microbe-associated molecular patterns (MAMPs) like LPS promotes a bacteria- or pathogen-dependent response program. Thus to fortify the intestinal barrier and to maintain intestinal homeostasis, epithelial cells express antimicrobial peptides (AMPs) as innate effectors molecules (5). Like vertebrates, insects are also able to secrete AMPs in the gut lumen and as insects lack an adaptive response it is a major component of host defense mechanism (28).

**Figure 4:**
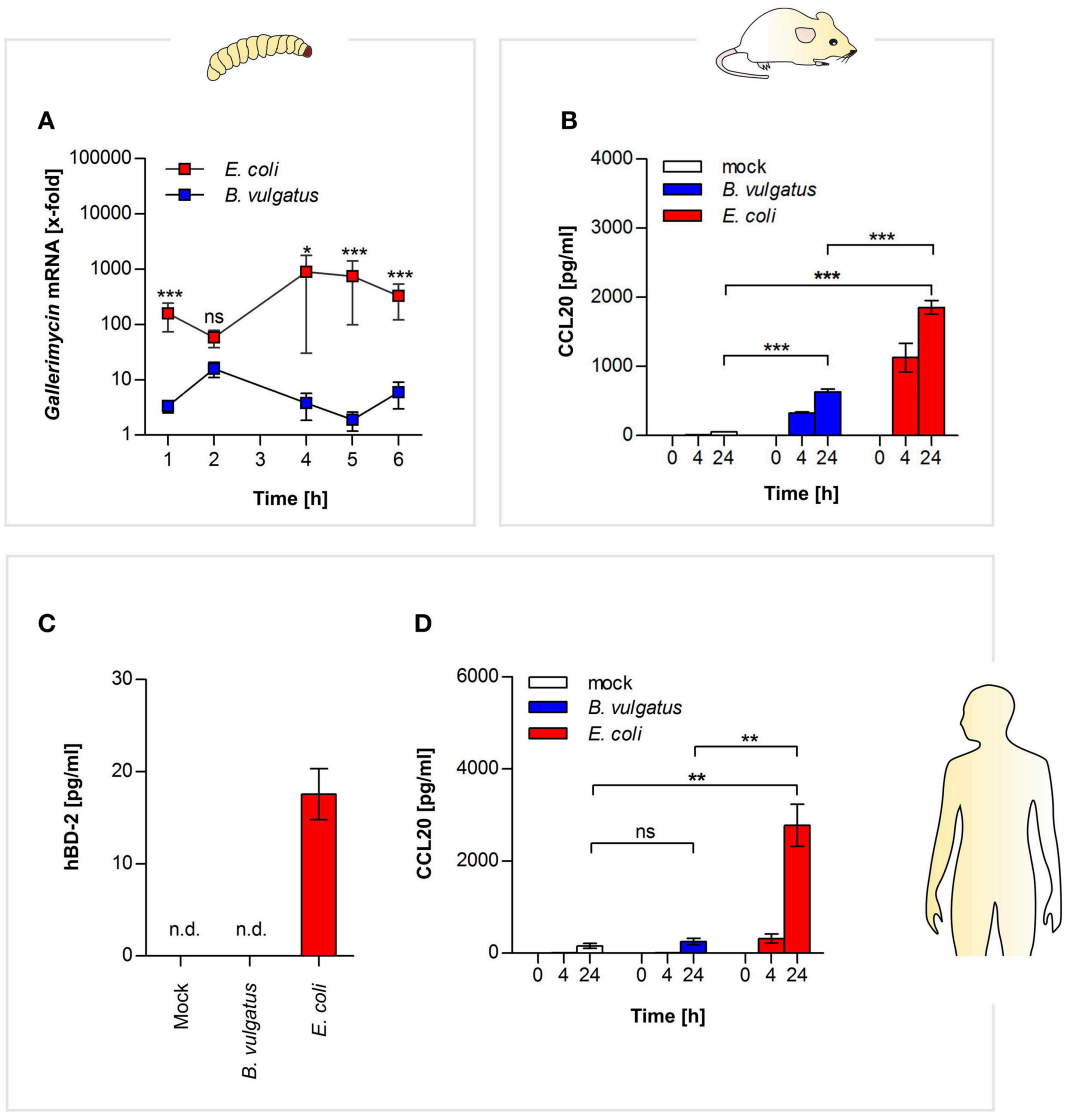
Commensal-induced antimicrobial responses in insect larvae, and mammalian epithelial cells. *Gallerimycin* gene expression was measured in RNA from *G. mellonella* larvae after *E. coli* or *B. vulgatus* oral administration (*n* = 8) **(A)**. Secretion of CCL20 was determined in mouse mICcl2 cell culture supernatants after commensal stimulation (*n* = 3) **(B)**. Human SW480 cells were stimulated with *E. coli* or *B. vulgatus* for 0, 4, and 24 h. hBD-2 **(C)** and human CCL20 **(D)** was measured by ELISA in culture supernatants (*n* = 3). Data represent the geometric means ± SEM. (n. d.: not detected).

We analyzed AMP production in *G. mellonella* and *Rag1*^−/−^ mice in response to oral administration of bacteria. In *G. mellonella* gene expression of defensin-like AMP gallerimycin was increased after 4 and 5 h in response to *E. coli* administration, whereas gene expression in *B. vulgatus*-administered larvae was significantly lower (Figure 4A). Interestingly, we observed a substantially decreasing bacterial load in *G. mellonella* larvae within 6 h after administration (Supplementary Figure S3). Additionally, we could also detect increased gene expression of other AMPs in *G. mellonella*, such as gloverin, cecropin, moricin, and lysozyme in response to *E. coli* administration (Supplementary Figures S4A–D). In summary, commensal-administered *G. mellonella* larvae produced a wide range of different antimicrobial peptides.

Similar responses could be observed in the mammalian host: in supernatants of *E. coli*-stimulated mouse small intestinal mICcl2 cells, higher secretion of CCL20 protein was detected compared to *B. vulgatus* stimulated cells (Figure 4B). As mentioned above, gallerimycin, BD-2, and CCL20 share structural and functional homology.

Therefore we additionally investigated if human colonic epithelial SW480 cells produce AMPs in response to symbiont or pathobiont stimulation after 0, 4, and 24 h. We could demonstrate that in contrast to B. vulgatus, stimulation with *E. coli* leads to secretion of human BD-2 after 24 h in cell culture supernatants (Figure 4C). Secretion of human CCL20 protein was measured by ELISA, and significant higher CCL20 was produced after 24 h in supernatants of *E. coli*-treated cells as compared to *B. vulgatus*-stimulated cells (Figure 4D).

In summary we provide evidence that symbionts or pathobionts trigger similar innate immune responses in both mammals and insects and that hence *G. mellonella* may be a suitable replace organism to distinguish mammalian intestinal symbionts form pathobionts.

## Discussion

In our study we present a comprehensive characterization of different innate immune mechanisms toward symbiotic and pathobiotic intestinal commensals in an invertebrate *G. mellonella* and vertebrate mouse model.

Previous *in silico* analysis of different components of innate immune responses, such as enzymes relevant in ROS signaling and AMP secretion, verified how closely related innate mechanisms among the models are and revealed evolutionary conservation of those genes involved in creating the responses. After *in silico* data generation, we created the hypothesis that in response to intestinal exposition of either a bacterial symbiont or pathobiont both vertebrates and invertebrates might activate comparable immune mechanisms. This hypothesis was successfully confirmed by our experimental data.

Both *G. mellonella*, as an insect representative, and mice, belonging to the mammalian class, are able to differentiate self from non-self and are capable to further discriminate between commensals. The ability to recognize foreign non-self elements is ancient and conserved among many primitive invertebrates (ameba), advanced invertebrates (sea stars) and of course among higher vertebrates like mice or rats (29). In order to react toward a non-self signal of potentially harmful invaders e.g., pathogens, hosts require PRRs that are able to sense microbial components–MAMPs–like LPS, peptidoglycan, flagellin, or lipoteichoic acid (LTA). The recognition of these MAMPs is a prerequisite for the initiation of appropriate follow-up mechanisms to eliminate potential intruders (30, 31). Innate immune responses are major contributors to acute inflammation induced by bacterial infection (32).

In mammals LPS released from Gram-negative bacteria can bind to the extracellular LPS-binding protein followed by binding to the CD14 molecule, a surface-expressed opsonic co-receptor. This reaction leads to the transfer of LPS to the MD2 accessory molecule and association with the TLR4 receptor, resulting in further immune signaling (31, 33). The upregulation of TLR4 receptor during intestinal inflammation was observed (10). Intestinal dendritic cells (DCs) have a key role in innate microbe recognition and influence adaptive immune responses (34). Intestinal DCs are decisive to differentiate between commensals and potential pathogens, and decide on maintenance of homeostasis or active immune responses (35). In previous work, we conveyed that *E. coli* and *B. vulgatus* mpk are differentially recognized by TLR2- and TLR4-overexpressing human embryonic kidney (HEK) cells. *E. coli* leads to much stronger stimulation of both TLR2 and 4 than *B. vulgatus* (36).

In *G. mellonella* two LPS-binding proteins were identified, which are considered to be PRRs (30). A highly abundant PRR in lepidopteran insects is apolipophorin which binds to LPS and triggers antimicrobial responses (30, 37). It is reported that *G. mellonella* manages to induce immune responses with differing intensities toward various microbes, which assumes also the differential recognition (38). However, all available information of *G. mellonella* MAMP recognition was obtained from studies involving systemic hemolymph infection. Until now, no evidence has been provided for differential intestinal recognition of Gram-negative microbes in *G. mellonella*. Interestingly, we also found upregulated hemolin gene expression, which is abundant in insect hemolymph and interacts with LPS and LTA (39). Hemolin a member of the immunoglobulin superfamily is also proposed to function as a recognition molecule (39). Both apolipophorin and hemolin work like opsonins (15). These results might suggest that certain bacterial compounds might cross the intestinal barrier and involve the hemolymph responses.

Insects and mammals share similar effectors that are secreted upon microbial challenge, such as production of ROS/RNS or AMPs (4, 29, 40), but only little is reported concerning insects and their immune responses toward intestinal microbial challenge. The majority of information available about insect intestinal immune responses toward microbes was obtained from *Drosophila*. The involvement of ROS responses to maintain intestinal homeostasis was recently reported in *Drosophila* (40). Xiao and colleagues showed that DUOX-related signaling was involved in balancing the load of the intestinal microbiota (40). DUOX belongs to the family of NADPH oxidases, which we could also identify in *G. mellonella* (41). These mentioned results are in agreement with our data generated in the present study. We could show that *G. mellonella* induces ROS- and RNS-related responses by *Nox-4* and *Nos* expression, which contributes to a decrease of bacterial load. Besides, we could provide evidence that *G. mellonella* induced differential ROS/RNS responses upon *E. coli* and *B. vulgatus* oral administration. The oxidative stress responses generated to clear the excessive bacteria was antagonized by induced *Gst* gene expression, which acts as an antioxidative molecule (6, 42). We assumed that the produced ROS and RNS were not intended to substantially harm the host, as the stress response was antagonized to retain cellular homeostasis. *G. mellonella* larvae have never been reported before to maintain gut homeostasis and preserve microbiota balance *via* intestinal epithelial cells. The *G. mellonella* model is widely used as a systemic infection model, in which ROS responses were determined in hemocytes after pathogen infection of the hemolymph (43).

Intestinal infections and inflammation in mammals are characterized by production of reactive oxygen and nitrogen species (44). ROS and RNS both similarly contribute to suppression of microbial growth and intestinal homeostasis in mammals (6). We were also able to detect elevated *Nos2* gene expression upon microbial challenge in mouse host cells using *in vitro* cell culture and *in vivo* in *Rag1*^−/−^ T cell-transplanted mice mono-associated with bacteria. Higher *Nos2* expression was found in response to *E. coli* stimulation. *Nos2* is highly expressed during pathogen infection and accumulates nitric oxide (NO) (45).NOS2 is mainly responsible for NO production, but it is further able to produce ROS, when concentrations of tetrahydrobiopterin or the co-substrate L-arginine is available in low quantities (6). *Rag1*^−/−^ mice are a well-established model for the influence of commensal bacteria on intestinal immune balance. The commensal induced innate immune response differs depending on the activation potential of the commensal and results in proliferation and polarization of T cells, which in turn might limit the commensal induced innate activation (46). Although here we used T cell transplanted *Rag1*^−/−^ mice we can observe innate immune responses comparable to that of *G. mellonella*, suggesting that there is no downregulation of the innate response by the transplanted T cells.

Some bacteria can overcome the oxidative stress responses, but can be eliminated by AMP responses, which serves as complementary second line antibacterial defense response (4, 41). We could show in both our invertebrate and in the mouse model that *E. coli* caused a higher production of AMPs compared to *B. vulgatus*. In *G. mellonella* all measured AMPs showed higher expression in *E. coli* administered larvae, which indeed points toward a distinct recognition and defined immune response according to different Gram-negative commensals. This is in line with the previous differential expression of PRRs in responses to those bacteria. We analyzed five out of 18 known AMPs from different AMP classes such as defensin-like peptides, cecropins, or moricins which are frequently investigated. When the *G. mellonella* immune system is challenged by a certain stimulus there is always a set of structurally and functionally distinct AMPs which is produced to enable a strong and effective response. A recent report showed that such AMP co-production along with the synergy of AMPs was an effective defense response due to potentiated antimicrobial effects (11).

Mice and humans secrete AMPs as well to maintain intestinal homeostasis. Different classes of antimicrobial peptides are secreted in the intestine such as C-type lectins, cathelicidins, or defensins. The latter two represent the most abundant types of AMPs (47). We were interested in the mammalian response of defensins, as similar molecules are also present in *G. mellonella*. The intestinal defensins are divided into α- and β-defensins and are at least in part dependent on innate PRRs to induce their transcription or secretion. α-defensins (HD5 and HD6) are produced by specialized Paneth cells whereas β-defensins are produced within different epithelial cells including enterocytes which contributes to a broader significance of β-defensins (10). BD-1 is constitutively expressed whereas BD-2, BD-3, and BD-4 show increased expression in response to pathogen attack (5). BD-2 and the antimicrobial chemokine CCL20 share structural homology and are able to bind to the CCR6 receptor. CCR6-CCL20 and CCR6-BD-2 respectively, play an important role in maintenance of intestinal homeostasis and restrict overgrowth of commensal bacteria (5, 48).

Using *in vitro* mouse small intestinal cells we could determine increased amounts of CCL20 after *E. coli* stimulation. We could confirm these results in human epithelial cells and even detect low amounts of the structurally similar AMP hBD-2 after stimulation with *E. coli*. AMP production is TLR-dependent and hBD-2 accumulates after TLR2, TLR3, and TLR4 recognition (5). It was shown that both hBD-2 and CCL20 were induced upon Caco-2 cell infection with EPEC, and are highly abundant in IBD patients (5). We propose that the high accumulation of both antimicrobial molecules might also be the consequence of the increased amounts of *E. coli* during the infection and inflammation, and its potential to stimulate TLR4 than only the inflammatory conditions.

Interpretation of our data suggests that the monitored immune reactions occur in different sequential timeframes and can be divided into faster and later responses: ROS- and RNS-related gene expression was upregulated quite early between 2 and 3 h, and antagonized by GST after 5 h, whereas the secretion of AMPs was induced only after oxidative stress responses have been completed. Thus, bacterial recognition must have occurred previously, since expression of apolipophorin, which recognizes and interacts with LPS structures, was induced already after 2 h.

**Figure 5:**
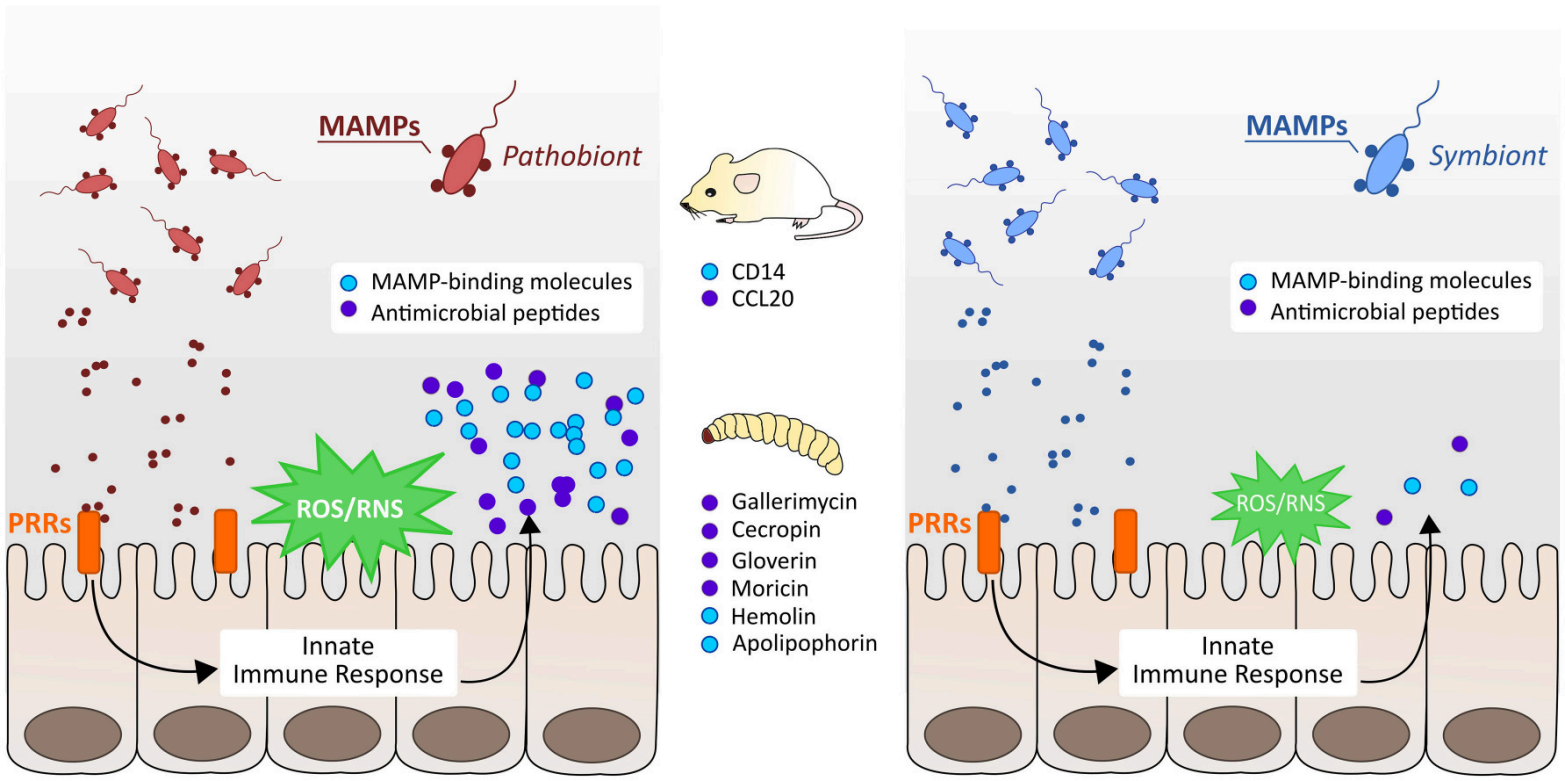
Schematic overview of vertebrate and invertebrate innate immune responses to symbiotic and pathobiotic bacteria. In both mice and *G. mellonella* larvae, pathobionts cause strong immune responses by the production of MAMP-binding recognition proteins, induction of reactive oxygen and nitrogen species (ROS/RNS), and secretion of different antimicrobial peptides (AMPs) (left). Symbionts cause only less pronounced immune responses and induce only low expression of immunity markers (right).

For the first time we provide evidence that *G. mellonella* recognizes commensals or their MAMPs by intestinal PRRs, and triggers effector molecules to cause oxidative stress responses, as well as the production of AMPs. Further we could uncover hints for differential intestinal commensal recognition that allow *G. mellonella* for discrimination of Gram-negative symbionts from pathobionts. Administration of the symbiont *B. vulgatus* leads to less pronounced immune responses (Figure 5, right) whereas administration of *E. coli* initiates stronger immune responses (Figure 5, left). Nevertheless, *E. coli* is not necessarily a pathobiont in *G. mellonella*, but still *E. coli* and *B. vulgatus* caused immune responses with different intensities and show a different immunogenic potential. The findings obtained from insects were therefore comparable to data obtained from vertebrates. We conclude that intestinal innate immune responses among *G. mellonella* and mammals are evolutionary conserved in response to challenge with intestinal commensals. Our results indicate that invertebrates can initiate proper immune responses to excessive loads of microbes and clear bacterial dysbiosis.

Symbionts and pathobionts and their recognition play crucial roles in intestinal homeostasis (2) and with our invertebrate *G. mellonella* model we provide an alternative model to screen commensals for their immunogenic potential. Hence we suggest *G. mellonella* as an invertebrate replacement model to discriminate intestinal commensals according to their immunogenic potential. Our future work will aim on the investigation of further microbes and their potential to activate innate immune responses via the intestinal tract in the insect model *G. mellonella*.

## Ethics statement

This study was carried out in accordance with the principles of the Basel Declaration. Protocols and experiments involving mice were reviewed and approved by the responsible Institutional Review Committee and the local authorities within H1/15 approval.

The work involving invertebrates does not need ethical permission according to German law.

## Author contributions

AL, ASt, and J-SF conceived and designed the experiments. AL, AS, AB, and RP performed the experiments. AL, ASt, and J-SF analyzed the data. SB performed the protein alignments. AL, ASt, and J-SF wrote the manuscript. All authors gave final approval to publish the article.

### Conflict of interest statement

The authors declare that the research was conducted in the absence of any commercial or financial relationships that could be construed as a potential conflict of interest.
