# Supplementary material for: Galleria mellonella: A Novel Invertebrate Model to Distinguish Intestinal Symbionts From Pathobionts
Source: Front Immunol. 2018 Sep 19;9:2114. doi: 10.3389/fimmu.2018.02114 (PMC6156133; doi:10.3389/fimmu.2018.02114)
Supplement: Supplementary file 2 [file Table_2.DOCX]

**Supplementary Table S2: Primers used in this study**

| **Primer name** | **Sequence 5´-3´** | **Reference** |
| --- | --- | --- |
| Gallerimycin f | GAAGTCTACAGAATCACACGA | This study |
| Gallerimycin r | ATCGAAGACATTGACATCCA |  |
| ubiquitin f | TCAATGCAAGTAGTCCGGTTC | Virulence (2014) 5: (4) 547-554 |
| ubiquitin r | CCAGTCTGCTGCTGATAAACC |  |
| Apo III f | AGACTTGCACGCCATCAAGA | This study |
| Apo III r | TGCATGCTGTTTGTCACTGC |  |
| Gloverin f | GTGTTGAGCCCGTATGGGAA | This study |
| Gloverin r | CCGTGCATCTGCTTGCTAAC |  |
| GST1 f | GACAGAAGTCCTCCGGTCAG | This study |
| GST r | TCCGTCTTCAAGCAAAGGCA |  |
| Lysozyme f | GGACTGGTCCGAGCACTTAG | This study |
| Lysozyme r | CGCATTTAGAGGCAACCGTG |  |
| Cecropin f | CTGTTCGTGTTCGCTTGTGT | This study |
| Cecropin r | GTAGCTGCTTCGCCTACCAC |  |
| Moricin f | GCTGTACTCGCTGCACTGAT | This study |
| Moricin r | TGGCGATCATTGCCCTCTTT |  |
| Hemolin f | CTCCCTCACGGAGGACAAAC | This study |
| Hemolin r | GCCACGCACATGTATTCACC |  |
| NOX-4 f | TGGCACGGCATCAGTTATCA | This study |
| NOX-4 r | ACAGCGACTGTCATGTGGAA |  |
| NOS f | ATGAAGGTGCTGAAGTCACAA | This study |
| NOS r | GCCATTTTACAATCGCCACAA |  |
| NOS2 f | GTTCTCAGCCCAACAATACAAGA | This study |
| NOS2 r | GTGGACGGGTCGATGTCAC |  |
| GSTP1 f | CGGCAAATATGTCACCCTCAT | This study |
| GSTP1 r | GCCAGGACTTGGTGGATCAG |  |
| CD14 f | GACCATGGAGCGTGTGCTTG | This study |
| CD14 r | GGACCAATCTGGCTTCGGAT |  |
| β-Actin f | CCCTGTGCTGCTCACCGA | This study |
| β-Actin r | ACAGTGTGGGTGACCCCGTC |  |
