# Supplementary material for: Galleria mellonella: A Novel Invertebrate Model to Distinguish Intestinal Symbionts From Pathobionts
Source: Front Immunol. 2018 Sep 19;9:2114. doi: 10.3389/fimmu.2018.02114 (PMC6156133; doi:10.3389/fimmu.2018.02114)
Supplement: Supplementary file 3 [file Table_3.DOCX]

**Supplementary Table S1: BLASTX analysis of glutathione S-transferase (GST) transcripts**

| ***G. mellonella*** | ***Mus musculus*** | | | | ***Homo sapiens*** | | | |
| --- | --- | --- | --- | --- | --- | --- | --- | --- |
| **Transcript** | **BLAST Hit** | **Identity** | **Query coverage** | **E value** | **BLAST Hit** | **Identity** | **Query coverage** | **E value** |
| GST1 | GST theta | 27% | 47% | 1x10^-12^ | GST  theta-1 | 31% | 36% | 3x10^-14^ |
| GST2 | GST theta-3 | 31% | 59% | 2x10^-18^ | GST  theta-4 | 33% | 57% | 9x10^-20^ |
| GST3 | GST theta-4 | 34% | 39% | 1x10^-12^ | GST  theta-2 | 34% | 41% | 3x10^-11^ |
| GST4 | GST Mu 4 | 27% | 86% | 2x10^-13^ | GST M2-3 | 26% | 87% | 1x10^-13^ |
| GST5 | GST omega-1 | 38% | 68% | 2x10^-45^ | GST Omega 1 | 39% | 68% | 4x10^-46^ |
| GST6 | GST omega-1 | 33% | 68% | 4x10^-35^ | GSTO1 | 36% | 71% | 1x10^-38^ |
| GST7 | GST  theta-3 | 28% | 70% | 9x10^-25^ | GST T1 | 33% | 69% | 1x10^-23^ |
| GST8 | GST  theta-3 | 38% | 48% | 5x10^-41^ | GST  theta 1 | 38% | 48% | 2x10^-40^ |
| GST9 | GST  theta-2 | 30% | 27% | 1x10^-19^ | GST  theta-1 | 29% | 25% | 1x10^-17^ |
| GST10 | microsomal GST 1 | 41% | 48% | 3x10^-27^ | microsomal GST 1 | 43% | 48% | 1x10^-26^ |
| GST11 | GST  theta 3 | 28% | 71% | 2x10^-21^ | GST theta-2 | 34% | 68% | 1x10^-22^ |
| GST12 | GST  theta-4 | 39% | 23% | 8x10^-11^ | GST theta-1 | 34% | 25% | 3x10^-09^ |
| GST13 | GST | 29% | 57% | 5x10^-19^ | GST A5 | 30% | 56% | 8x10^-15^ |
| GST14 | GST  theta-4 | 29% | 68% | 3x10^-20^ | GSTT2 | 30% | 87% | 6x10^-23^ |
| GST15 | no match | - | - | - | no match | - | - | - |
| GST16 | GST theta | 27% | 77% | 3x10^-15^ | GST | 31% | 67% | 6x10^-15^ |
| GST17 | GST theta | 29% | 71% | 2x10^-24^ | GST T1 | 30% | 71% | 1x10^-23^ |
| GST18 | GST Zeta 1-1 | 47% | 66% | 2x10^-59^ | GST zeta 1 | 45% | 65% | 5x10^-56^ |
| GST19 | GST theta | 30% | 67% | 3x10^-18^ | GST T1 | 33% | 67% | 1x10^-17^ |
