# Supplementary figures and images for: Galleria mellonella: A Novel Invertebrate Model to Distinguish Intestinal Symbionts From Pathobionts
Source: Front Immunol. 2018 Sep 19;9:2114. doi: 10.3389/fimmu.2018.02114 (PMC6156133; doi:10.3389/fimmu.2018.02114)

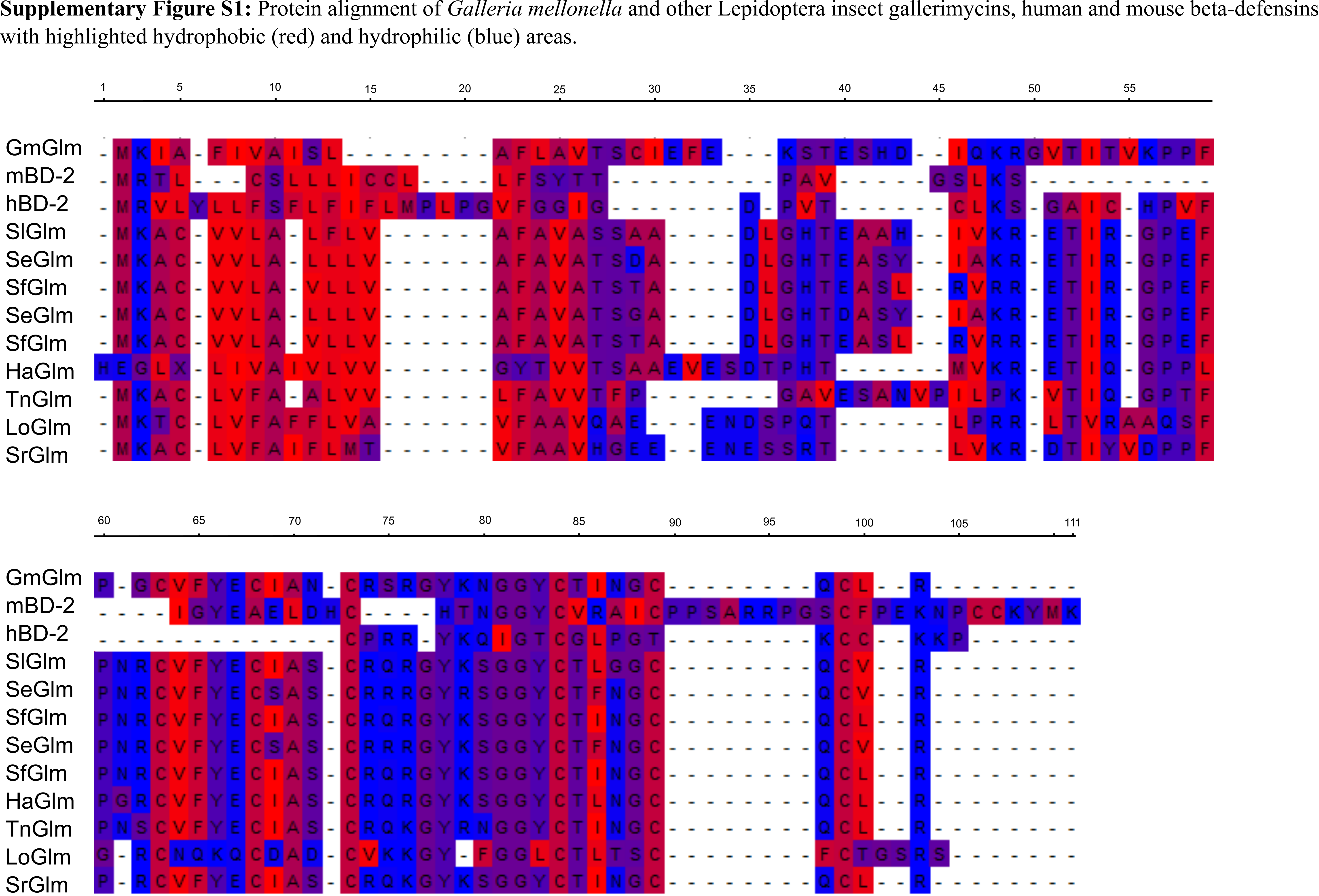

Supplement: Supplementary file 4 [file Image_1.PNG]

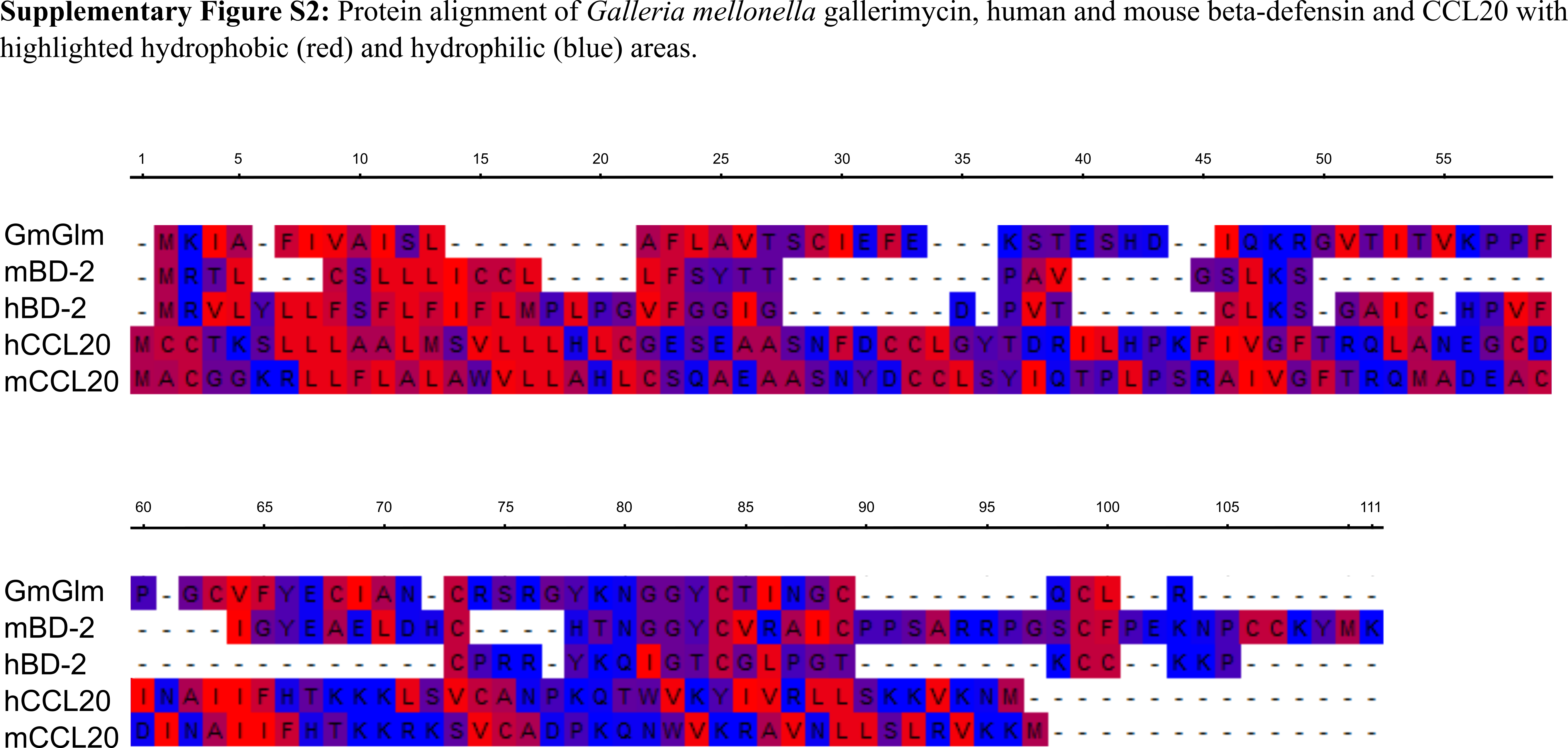

Supplement: Supplementary file 5 [file Image_2.PNG]

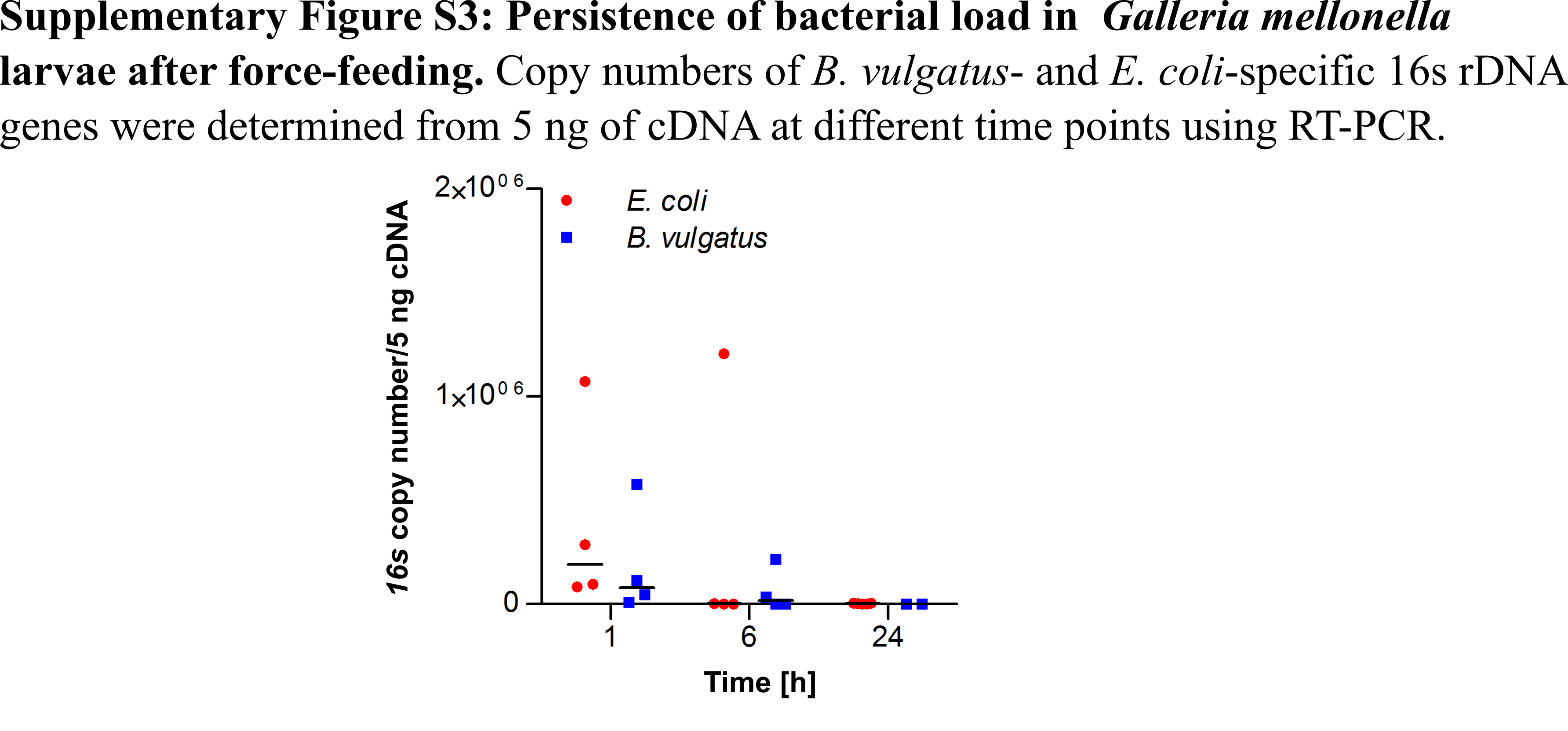

Supplement: Supplementary file 6 [file Image_3.PNG]

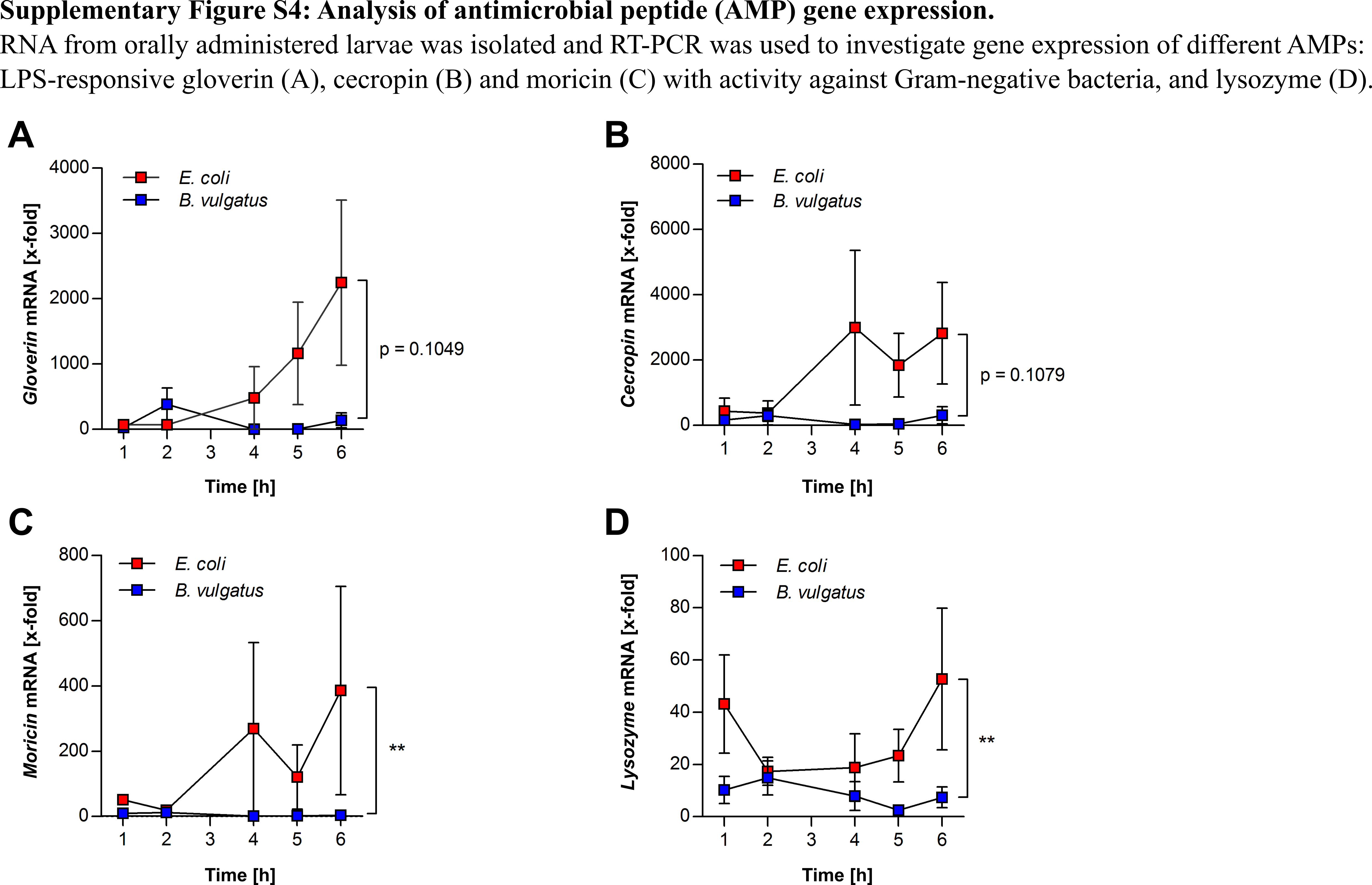

Supplement: Supplementary file 7 [file Image_4.PNG]
